# Is cognition integral to psychopathology? A population-based cohort study

**DOI:** 10.1017/S0033291723000934

**Published:** 2023-11

**Authors:** Anat Rotstein, Suzanne Fund, Stephen Z. Levine, Abraham Reichenberg, Judy Goldenberg

**Affiliations:** 1 Department of Psychiatry, Icahn School of Medicine at Mount Sinai, New York, New York, USA; 2 Department of Gerontology, University of Haifa, Haifa, Israel; 3 Department of Behavioral Sciences, Israel Defense Forces, Israel; 4 School of Public Health, University of Haifa, Haifa, Israel; 5 Department of Environmental Medicine & Public Health, Icahn School of Medicine at Mount Sinai, New York, New York, USA

**Keywords:** Adolescents, Brief symptom inventory, Intellectual performance, Intelligence, Psychopathological symptoms, Structural models

## Abstract

**Background:**

Lower cognitive functioning has been documented across psychiatric disorders and hypothesized to be a core deficit of mental disorders. Situating psychopathology and cognition as part of a unitary construct is therefore important to understanding the etiology of psychiatric disorders. The current study aims to test competing structural models of psychopathology and cognition in a large national cohort of adolescents.

**Methods:**

The analytic sample consisted of 1189 participants aged 16–17 years, screened by the Israeli Draft Board. Psychopathology was assessed using a modified version of the Brief Symptom Inventory, and cognition was assessed based on four standardized test scores ((1) mathematical reasoning, concentration, and concept manipulation; (2) visual-spatial problem-solving skills and nonverbal abstract reasoning; (3) verbal understanding; (4) categorization and verbal abstraction). Confirmatory factor analysis was implemented to compare competing structural models of psychopathology with and without cognition. Sensitivity analyses examined the models in different subpopulations.

**Results:**

Confirmatory factor analysis indicated a better model fit of psychopathological symptoms without cognition (RMSEA = 0.037; TLI = 0.991; CFI = 0.992) than with cognition (RMSEA = 0.04–0.042; TLI = 0.987–0.988; CFI = 0.988–0.989). Sensitivity analyses supported the robustness of these results with a single exception. Among participants with low cognitive abilities (*N* = 139), models that integrated psychopathological symptoms with cognition had a better fit compared to models of psychopathology without cognition.

**Conclusions:**

The current study suggests that cognition and psychopathology are, generally, independent constructs. However, within low cognitive abilities, cognition was integral to the structure of psychopathology. Our results point toward an increased vulnerability to psychopathology in individuals with low cognitive abilities and may provide valuable information for clinicians.

## Introduction

Converging evidence indicates that psychopathology and cognitive dysfunction often coexist. Indeed, the Diagnostic and Statistical Manual of Mental Disorders (DSM-5-TR, American Psychiatric Association, 2022) states that psychopathology is characterized by clinically significant disturbances of emotion regulation, behavior, or cognition. Recent psychiatric conceptualizations have suggested that impaired cognitive ability may play a core role in psychopathology (Harvey et al., 2022; Kahn, 2020; Moura et al., 2021; Tripoli et al., 2021). Large population-based studies have supported this notion showing cognitive impairments across psychiatric disorders, although with different manifestations. For instance, the extent and timing of cognitive deficits in schizophrenia differ between cognitive domains [e.g. processing speed deficits appear early in life, while deficits in working and verbal memory may only appear later (Fett, Reichenberg, & Velthorst, 2022)]. Depressed individuals exhibit impairments in executive function and attention throughout the course of the disorder, including in remission (Rock, Roiser, Riedel, & Blackwell, 2014). Other psychopathological disorders present primarily impairment in domains of executive functioning, such as the executive component of the working memory system in anxiety (Eysenck, Derakshan, Santos, & Calvo, 2007), executive functioning and verbal memory in post-traumatic stress disorder (Polak, Witteveen, Reitsma, & Olff, 2012), and planning in borderline personality disorder (McClure, Hawes, & Dadds, 2016). The high rates of comorbidity between psychiatric disorders and cognitive impairments suggest that cognition may be integral to psychopathology. It is, however, unclear whether cognitive impairments are themselves part of a pathway to psychopathology, or merely indicate vulnerability (Reichenberg, 2005).

Still, contradictory evidence exists and the association between cognition and psychopathology remains a topic of debate. Low levels of intellectual performance are not always observed in psychiatric disorders (MacCabe et al., 2010, 2012; Oomen et al., 2021; Wraw, Deary, Der, & Gale, 2016), and medications that have efficacy in the treatment of psychopathological symptoms, do not affect the associated cognitive impairment (Kahn & Keefe, 2013; Keefe et al., 2013), suggesting independence of psychopathology and cognitive ability. Despite the interest in the structure of psychopathology, the hypothesis that cognition is integral to the structure of psychopathology has not been empirically tested except for the specific case of schizophrenia (Toulopoulou et al., 2007).

The current study examined whether cognition is integral to the structure of psychopathology using a large national population-based cohort of adolescents. Given that adolescence marks the onset of approximately 50% of all lifetime psychiatric disorders (Fusar-Poli et al., 2021; Paus, Keshavan, & Giedd, 2008), focusing on this population segment is particularly relevant to psychopathology research.

## Methods

### Study population and procedure

The source population consisted of Israeli adolescents aged 16–17, undergoing mandatory Israeli Draft Board screening (described in detail elsewhere; Weiser et al. 2004, 2007). The Draft Board screening is applied regardless of whether an individual is eligible for national service or not based on medical, psychiatric, or social grounds. All participants (*N* = 1374; average age of 16.34; s.d. = 0.48) were assessed on randomly selected days in 2017 (see online Supplementary Fig. 1 for study flow diagram). This study received ethical approval with a waiver of informed consent from the Institutional Review Board at the University of Haifa (Application no. 090/21).

### Psychopathological symptoms

Psychopathological symptoms were assessed using a modified version of the Brief Symptom Inventory (Derogatis & Melisaratos, 1983; Rotstein, Goldenberg, Fund, Levine, & Reichenberg, 2021). The inventory covers nine dimensions of psychopathology: psychoticism, paranoid ideation, depression, anxiety, phobic anxiety, somatization, obsession-compulsion, interpersonal sensitivity, hostility (i.e. conduct disorder), and substance abuse (i.e. alcohol and drug use). The latter was added by the Israeli Draft Board. Symptom ratings characterize the intensity of distress during the past month on a 5-point Likert scale from 0 (Not at all) to 4 (Extremely). The entire inventory took approximately 10–12 min to complete. Previous studies have shown this measure to have sufficient internal reliability (α = 0.95 for the general symptom severity index; average α = 0.71 for all subscales) and convergence validity (*r* = −0.62 for the general symptom severity index; average *r* = −0.49 for all subscales; Canetti, Shalev, & De-Nour, 1994).

### Cognition

Cognitive functioning was assessed using four progressive time-limited tests that measure: (1) Mathematical reasoning, concentration and concept manipulation; (2) Visual-spatial problem-solving skills and nonverbal abstract reasoning; (3) Verbal understanding (based on the ability to comprehend and perform verbal instructions); (4) Categorization and verbal abstraction. Test scores reflect the number of correct answers. The total score of this assessment was found valid when compared to the Wechsler Adult Intelligence Scale score (Wechsler, 1955; *r* > 0.90) and associated with external outcomes (i.e. rank upon discharge; *r* > 0.41; Gal, 1986). The assessment is described in detail elsewhere (Reichenberg et al., 2005).

### Analytic sample and data analyses

We excluded 185 individuals (13.5%) due to missing data on psychopathological symptoms and/or cognitive assessment scores (online Supplementary Fig. 1). Descriptive statistics of the association between psychopathological symptoms and cognitive ability were computed and visualized using continuous (total psychopathological symptoms scores; overall cognitive assessment scores) and categorical data (high standardized cognitive assessments scores: >115; low standardized cognitive assessments scores:< 85; average standardized cognitive assessments scores: >85, <115; high psychopathology: top 20% of the sample, based on total psychopathological symptom scores; low psychopathology: bottom 20% of the sample; average psychopathology: participants between the top 20% and bottom 20% of the sample).

**Figure 1.**
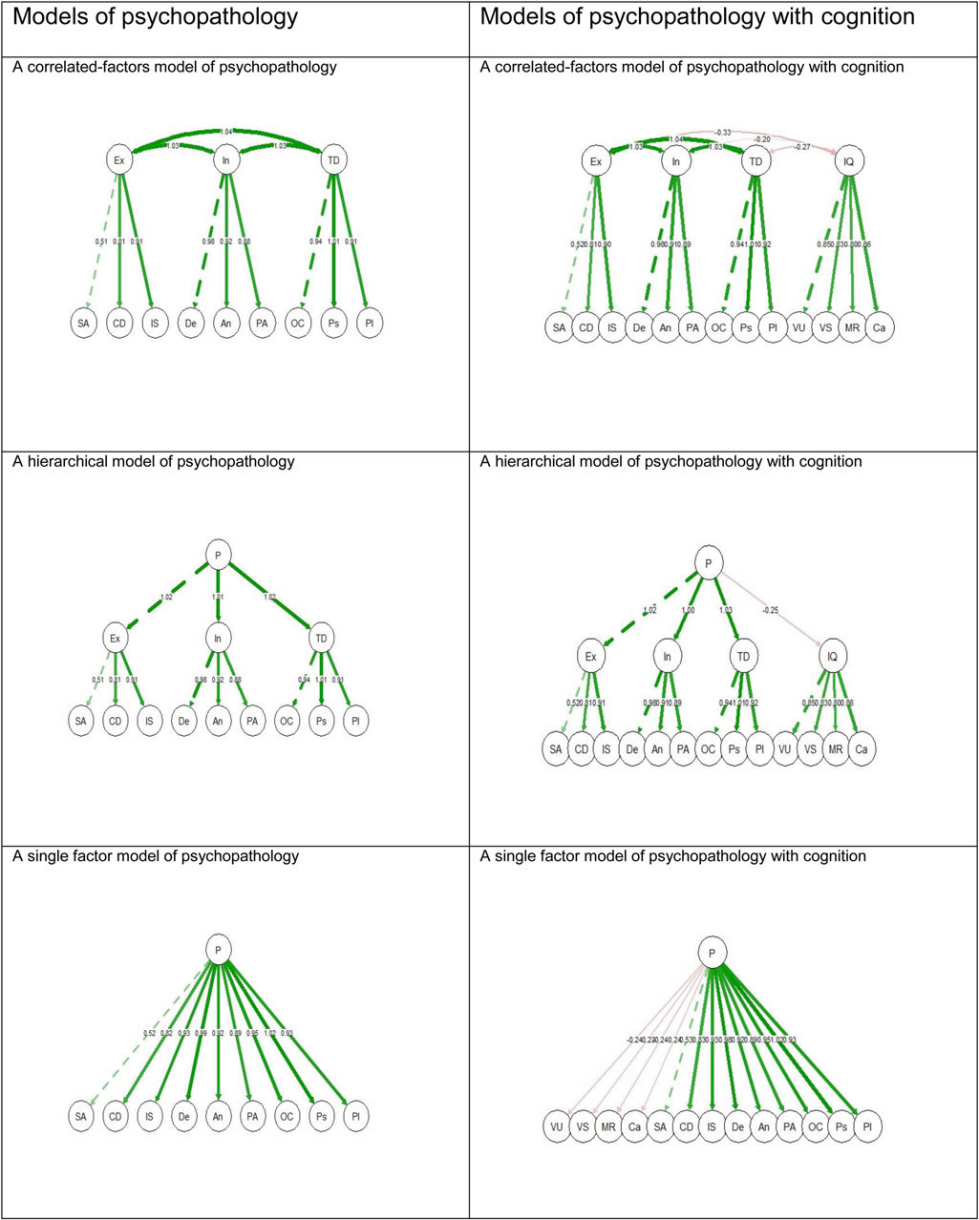
models of the structure of psychopathology with and without cognition. *Note.* Abbreviations. P, A general factor of psychopathology; Ex, Externalizing; In, Internalizing; TD, Thought Disorder; SA, Substance Abuse; CD, Conduct Disorder (i.e. Hostility); IS, Interpersonal Sensitivity; De, Depression; An, Anxiety; PA, Phobic Anxiety; OC, Obsessive-Compulsive, Ps, Psychoticism; PI, Paranoid Ideation; IQ, Intelligence Quotient; VU, Verbal Understanding; VS, Visual-Spatial problem-solving abilities; MR, Mathematical reasoning; Ca, Categorization abilities. The numbers represent the standardized loadings beta estimates. Green lines represent positive estimates, whereas red lines represent negative estimates. All factor loadings are statistically significant. Due to multicollinearity, the standardized loadings estimates can exceed the bounds of (−1,1) (Deegan, 1978).

The primary analysis consisted of fitting confirmatory factor analytic models to the entire cohort. Namely, three types of standard models of the structure of psychopathology were analyzed based on previous research (Caspi et al., 2014): A correlated-factor model, a hierarchical model, and a single factor model (see Fig. 1). These models display variations of one *general psychopathology dimension* (i.e. P) and three higher-order psychopathology factors (Caspi et al., 2014): an *internalizing* liability to depression and anxiety; an *externalizing* liability to antisocial and substance-use disorders; and a *thought disorder* liability to symptoms of psychosis. The externalizing higher-order psychopathology factor included the following dimensions of psychopathology: substance abuse, conduct disorder (i.e. hostility), and interpersonal sensitivity. The internalizing higher-order psychopathology factor included the following dimensions of psychopathology: depression, anxiety, and phobic anxiety. The thought disorder higher-order psychopathology factor included the following dimensions of psychopathology: obsessive-compulsive, psychoticism, and paranoid ideation. The somatization dimension of psychopathology was not used in the current study because previous evidence indicates it is a separate factor (Marek et al., 2020).

All three models were run twice. First, for psychopathological symptoms without cognition, then for psychopathological symptoms with cognition. Psychopathological symptoms were treated as ordered variables, whereas subscales of cognitive ability were treated as continuous. The models were implemented with the Weighted Least Squares Means variance adjusted estimator and the Yuan–Bentler test. These do not assume normally distributed variables and provide the best option for modeling ordered data (Brown, 2015). Fit indices of models with and without cognition were compared for each type of model. Specifically, the goodness of fit was determined with the Tucker–Lewis Index (TLI), where values over 0.95 represent good fit, the Comparative Fit Index (CFI), where values over 0.95 represent good fit, and the Root-Mean-Square Error of Approximation (RMSEA) where values under 0.05 represent good fit, similar to previous research of psychopathology (Caspi et al., 2014).

#### Sensitivity analyses

Sensitivity analyses were implemented to examine the models above in subpopulations with previously shown inconsistent associations between psychopathology and cognition. Specifically, we examined highly symptomatic participants (top 20% of the sample, based on total psychopathological symptom scores) because symptom severity was found related to cognitive performance (for a review, see Russo, Murray, & Reichenberg, 2013). We next focused on participants with low standardized cognitive assessment scores (< 85), because of previously found associations between low cognitive abilities and most psychiatric disorders (Weiser et al., 2021). We also separately investigated male and female participants due to evidence of disorder-specific, sex-specific cognitive differences (Zanelli et al., 2013). An additional sensitivity analysis was run on the entire cohort to examine psychopathology models based only on psychotic symptoms, on the grounds that schizophrenia and other psychoses are strongly associated with cognitive impairment (Zanelli et al., 2019). All analyses were conducted in R version 4.1.0.

## Results

### Sample characteristics

**Figure 2.**
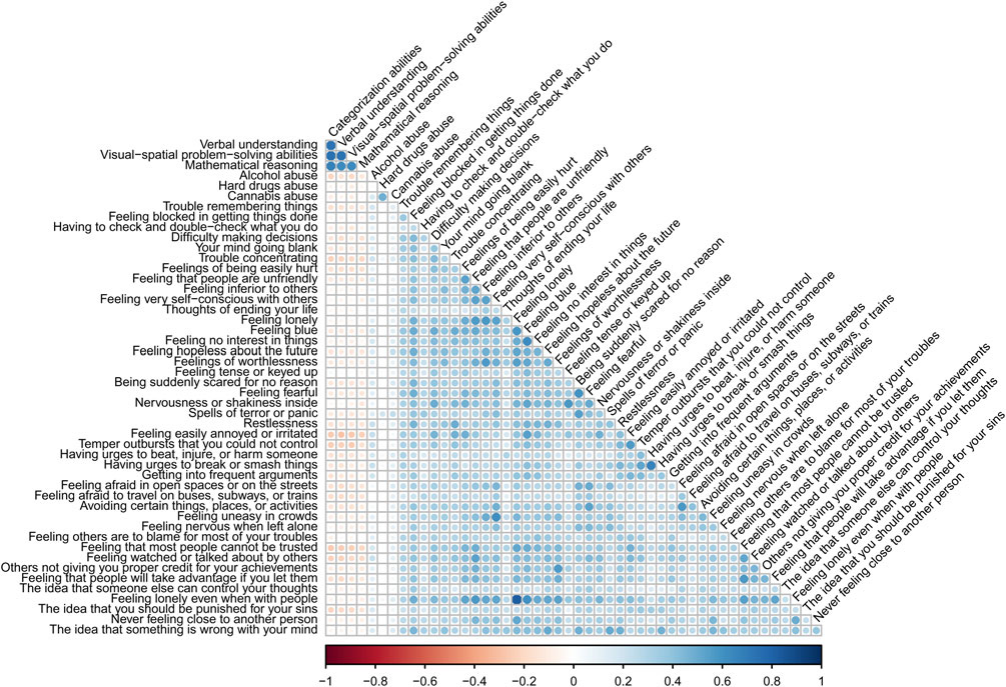
Bivariate Pearson correlations between the study variables.

**Figure 3.**
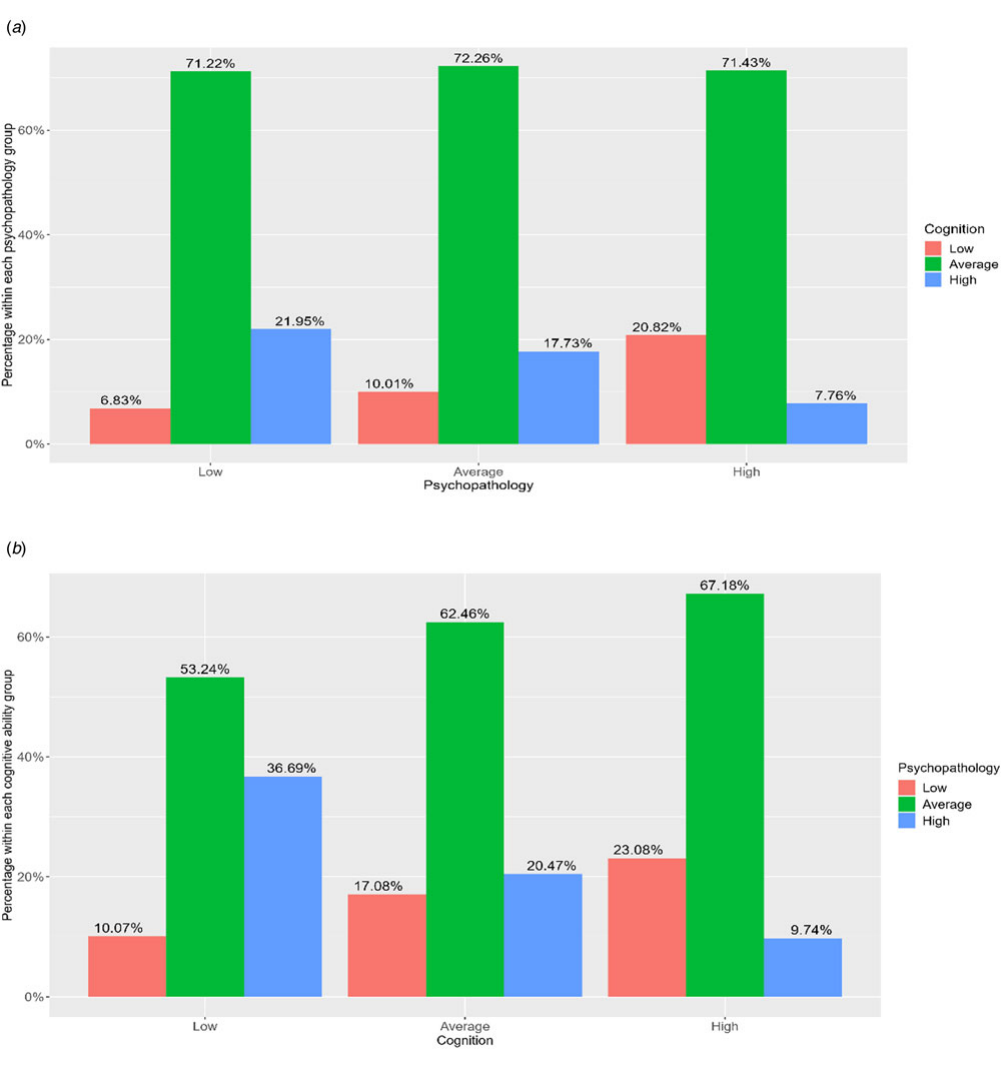
(a)The percentage of high, average, and low cognition for distinct levels of psychopathology. (b) The percentage of high, average, and low psychopathology for distinct levels of cognition.

The analytic sample (*N* = 1189) included 603 males and 586 females and had an average age of 16.32 (s.d. = 0.43) years. Sample characteristics are displayed in online Supplementary sTable 1. In general, higher cognitive ability was associated with lower psychopathology in both continuous (*r* = −0.23; *p* < 0.001) and categorical data (χ^2^ = 39.22; df = 4; *p* < 0.001). Bivariate Pearson correlations between the study variables are displayed in Fig. 2. Percentages of high, average, and low cognitive ability for distinct psychopathological groups as well as high, average, and low psychopathology for distinct cognitive ability groups are presented in Fig. 3.

### Is cognition integral to psychopathology?

Comparing the fit of three confirmatory factor analytic models (a correlated-factors model, a hierarchical model, and a single factor model) showed a better fit for psychopathological symptoms without cognition (RMSEA = 0.037; TLI = 0.991; CFI = 0.992), than for psychopathological symptoms with cognition (RMSEA = 0.04–0.042; TLI = 0.987–0.988; CFI = 0.988–0.989). Specifically, the RMSEA was lower, and the TLI and CFI were higher for a correlated-factors model, a hierarchical model, and a single factor model of psychopathology without cognition. Note that these three confirmatory factor analytic models of psychopathology fit our data similarly well, with the single factor model offering a slightly more parsimonious solution. Fit indices are presented in Table 1. Factor loadings are presented in online Supplementary sTables 2–7.

**Table 1.** Fit indices for models of psychopathology with and without cognition

Model	RMSEA	TLI	CFI
Entire cohort (*N* = 1189)			
A correlated-factors model of psychopathology without cognition	0.037	0.991	0.992
A correlated-factors model of psychopathology with cognition	0.040	0.988	0.989
A hierarchical model of psychopathology without cognition	0.037	0.991	0.992
A hierarchical model of psychopathology with cognition	0.042	0.987	0.988
A single factor model of psychopathology without cognition	0.037	0.991	0.992
A single factor model of psychopathology with cognition	0.040	0.988	0.989
Participants with low cognitive abilities (standardized cognitive assessments scores < 85; *N* = 139)			
A correlated-factors model of psychopathology without cognition	0.070	0.980	0.981
A correlated-factors model of psychopathology with cognition	0.067	0.978	0.979
A hierarchical model of psychopathology without cognition	0.070	0.980	0.981
A hierarchical model of psychopathology with cognition	0.066	0.979	0.980
A single factor model of psychopathology without cognition	0.070	0.980	0.981
A single factor model of psychopathology with cognition	0.068	0.977	0.979

*Note.* The goodness of fit was determined by the Tucker–Lewis Index (TLI) where values over 0.95 represent good fit, the Comparative Fit Index (CFI) where values over 0.95 represent good fit, and the Root-Mean-Square Error of Approximation (RMSEA), where values under 0.05 represent good fit, similar to previous research of psychopathology (Caspi et al., 2014). For all models fitted to the entire cohort, RMSEA P-value = 1; For all models fitted to participants with low cognitive abilities, RMSEA P-value = 0.

#### Sensitivity analyses

Generally, sensitivity analyses of the three models showed a better fit for psychopathological symptoms without cognition compared to the structure where cognition is integral to psychopathology (fit indices are presented in online Supplementary sTable 8). This was the case in highly symptomatic participants; in both males and females; and in psychopathology models based only on psychotic symptoms. In contrast, for participants with low standardized cognitive assessments scores (equivalent to < 85, *N* = 139), the models of psychopathological symptoms with cognition showed a better fit than the models without cognition (Table 2).

## Discussion

Using a large national cohort of adolescents, the current study examined whether cognition is integral to the structure of psychopathology. The results showed that the structure of psychopathology is better outlined independently to cognitive functioning. This result was robust across different models of psychopathology (e.g. with and without a general factor of psychopathology), and in multiple subpopulations.

The current study results are inconsistent with recent conceptualizations of schizophrenia (Harvey et al., 2022; Kahn, 2020; Moura et al., 2021; Tripoli et al., 2021). Cognitive impairments represent one of the core features of schizophrenia and have been considered of great relevance since the earliest conceptualizations of the disorder (Kahn, 2020). Cognitive impairments are as prevalent as delusions, hallucinations, or thought disorders, and are present even before the development of full-blown psychosis (Davidson, 2019). Impaired cognition is persistent rather than intermittent (Davidson, 2019) and has a substantial negative impact on functional and recovery outcomes (Harvey et al., 2022).

Still, the results are consistent with prior findings of an overall pattern of weak correlations between psychopathology and cognition in the general population (<0.2; Caspi *et al*. 2014; Southward, Cheavens, & Coccaro, 2022). Our results are also consistent with recent research on high-risk samples that showed independence of cognition from psychopathology (Littlefield, Lane, Gette, Watts, & Sher, 2021; Southward et al., 2022). We extend these studies by using an unselected national sample and testing the structure of psychopathology within relevant sub-groups.

In our primary analysis, as well as in most sensitivity analyses (including models of highly symptomatic participants), the addition of cognition did not improve the fit of the models representing the structure of psychopathology. A single exception was of models fitted to individuals with low cognitive abilities, in which psychopathology and cognition were better represented as a single integrated construct. The integration of psychopathology and cognition suggests that cognition may not only function as a possible marker for early detection and prevention of psychiatric illness (Harvey et al., 2022) but may also contribute substantially to other deficits and poor functional outcomes (Harvey & Strassnig, 2019). Nevertheless, this may also suggest that the observed association between lower cognitive functioning and higher psychopathology across psychiatric disorders is mostly due to an epiphenomenon and/or other shared etiological factors (e.g. Harvey, Koren, Reichenberg, & Bowie, 2006; Reichenberg, 2005).

### Limitations

The current study has some limitations. Psychopathology was not defined based on psychiatric diagnoses, unlike some previous studies (e.g. Rock et al., 2014). Future studies may investigate the association between cognition and psychopathology based on medical diagnoses of mental disorders. In our study, psychopathology was measured with the Brief Symptom Inventory (Derogatis & Melisaratos, 1983), a screener based on symptoms reported in the last month. While this reporting timeframe may underestimate the prevalence of psychopathology, it may simultaneously limit recall biases. Underestimation or overestimation may also occur due to motivational factors that may affect both psychopathology and cognition, as these were assessed in the context of evaluation and selection. Also, our data is cross-sectional and causal inference is limited. Although most research supports the hypothesis that low cognitive abilities are a risk factor for increased psychopathology, intellectual performance may change post-onset (e.g. patients with psychoses experience cognitive decline after illness onset; Zanelli et al., 2019). Furthermore, cross-sectional data does not account for variations occurring naturally over time in psychopathological measures. Therefore, whether the results would replicate longitudinally or with other measures is unclear. However, our study is based on an inventory that is well validated and has high reliability (Canetti et al., 1994), thereby supporting the validity of our results. In terms of generalizability, this study is based on a national sample, yet it focuses on adolescents. While adolescence marks the onset of approximately 50% of all lifetime psychiatric disorders (Fusar-Poli et al., 2021; Paus et al., 2008), our limited age range may not reflect the age at which different psychiatric disorders typically emerge and the results may vary in other age groups. Finally, our subsample of individuals with low cognitive abilities was small and likely underpowered. Future studies may examine larger samples.

### Conclusions and implications

In summary, based on a large national sample, the current study results suggest that in late adolescence, cognition and psychopathology are, generally, independent constructs. However, within low cognitive abilities, integration of psychopathology and cognition was warranted. Therefore, our results point toward an increased vulnerability to psychopathology in individuals with low cognitive abilities. Although our study implies that impaired cognition may provide valuable diagnostic information for clinicians, it remains unclear whether cognitive impairment is part of a causal pathway to psychopathology.

## Supporting information

Rotstein et al. supplementary material Rotstein et al. supplementary material

## Supplementary material

The supplementary material for this article can be found at https://doi.org/10.1017/S0033291723000934
