# Supplementary material for: Is cognition integral to psychopathology? A population-based cohort study
Source: Psychol Med. 2023 Apr 28;53(15):7350–7. doi: 10.1017/S0033291723000934 (PMC10719669; doi:10.1017/S0033291723000934)
Supplement: Rotstein et al. supplementary material [file S0033291723000934sup001.pdf]

Supplementary Material

Is Cognition Integral to Psychopathology?

A Population-Based Cohort Study

Anat Rotstein[1,2]

Suzanne Fund[3]

Stephen Z. Levine[4]

Abraham Reichenberg[1,5]

Judy Goldenberg[3]

1. Department of Psychiatry, Icahn School of Medicine at Mount Sinai, New York, New York.
2. Department of Gerontology, University of Haifa, Israel.
3. Department of Behavioral Sciences, Israel Defense Forces, Israel.
4. School of Public Health, University of Haifa, Israel.
5. Department of Environmental Medicine & Public Health, Icahn School of Medicine at Mount Sinai, New York, New York.

Contents

sFigure 1: Study flow diagram 2

sTable 1: Sample characteristics 3

sTable 2: Factor loadings for a correlated-factors model of psychopathology 5

sTable 3: Factor loadings for a correlated-factors model of psychopathology with cognition 6

sTable 4: Factor loadings for a hierarchical model of psychopathology 7

sTable 5: Factor loadings for a hierarchical model of psychopathology with cognition 8

sTable 6: Factor loadings for a single factor model of psychopathology 9

sTable 7: Factor loadings for a single factor model of psychopathology with cognition 10

sTable 8: Fit indices for models of psychopathology with and without cognition: Sensitivity analysis of subpopulations 11

References 12

sFigure 1: Study flow diagram

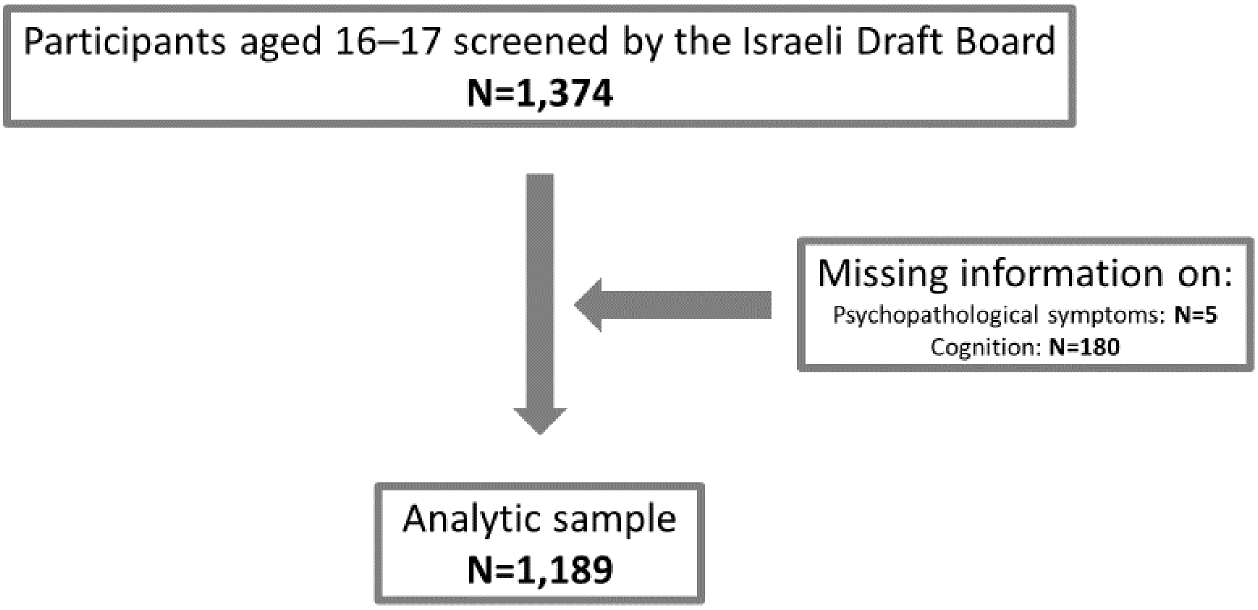

sTable 1: Sample characteristics

	Mean (SD)
**Cognition**	
Categorization abilities	15.66 (3.51)
Verbal understanding	20.91 (5.73)
Visual-spatial problem-solving abilities	22.49 (5.59)
Mathematical reasoning	13.18 (5.53)
**Psychopathology**	
Alcohol abuse	0.27 (0.56)
Hard drugs abuse	0.01 (0.13)
Cannabis abuse	0.04 (0.24)
Trouble remembering things	0.27 (0.70)
Feeling blocked in getting things done	0.37 (0.76)
Having to check and double-check what you do	0.15 (0.58)
Difficulty making decisions	0.68 (0.95)
Your mind going blank	0.19 (0.56)
Trouble concentrating	0.91 (1.14)
Feelings of being easily hurt	0.47 (0.90)
Feeling that people are unfriendly	0.39 (0.78)
Feeling inferior to others	0.18 (0.54)
Feeling very self-conscious with others	0.23 (0.62)
Thoughts of ending your life	0.04 (0.27)
Feeling lonely	0.35 (0.76)
Feeling blue	0.73 (0.95)
Feeling no interest in things	0.63 (0.97)
Feeling hopeless about the future	0.29 (0.72)
Feelings of worthlessness	0.25 (0.71)
Feeling tense or keyed up	1.10 (1.05)
Being suddenly scared for no reason	0.17 (0.55)
Feeling fearful	0.28 (0.71)
Nervousness or shakiness inside	0.67 (0.96)
Spells of terror or panic	0.14 (0.58)

Restlessness	0.33 (0.77)
Feeling easily annoyed or irritated	0.89 (1.08)
Temper outbursts that you could not control	0.24 (0.69)
Having urges to beat, injure, or harm someone	0.12 (0.50)
Having urges to break or smash things	0.15 (0.57)
Getting into frequent arguments	0.29 (0.66)
Feeling afraid in open spaces or on the streets	0.14 (0.52)
Feeling afraid to travel on buses, subways, or trains	0.17 (0.62)
Avoiding certain things, places, or activities	0.20 (0.63)
Feeling uneasy in crowds	0.16 (0.55)
Feeling nervous when left alone	0.18 (0.56)
Feeling others are to blame for most of your troubles	0.23 (0.58)
Feeling that most people cannot be trusted	0.63 (1.05)
Feeling watched or talked about by others	0.46 (0.86)
Others not giving you proper credit for your achievements	0.56 (0.93)
Feeling that people will take advantage if you let them	0.38 (0.86)
The idea that someone else can control your thoughts	0.06 (0.31)
Feeling lonely even when with people	0.42 (0.86)
The idea that you should be punished for your sins	0.20 (0.61)
Never feeling close to another person	0.27 (0.65)
The idea that something is wrong with your mind	0.15 (0.57)

Note.

Abbreviations. SD: Standard Deviation

sTable 2: Factor loadings for a correlated-factors model of psychopathology

Factor loadings

	Ex	In	TD
SA	.512	0	0
CD	.808	0	0
IS	.911	0	0
De	0	.978	0
An	0	.915	0
PA	0	.884	0
OC	0	0	.941
Ps	0	0	1.009
PI	0	0	.914

Note.

Abbreviations. Ex: Externalizing; In: Internalizing; TD: Thought Disorder; SA: Substance Abuse; CD: Conduct Disorder (i.e., Hostility); IS: Interpersonal Sensitivity; De: Depression; An: Anxiety; PA: Phobic Anxiety; OC: Obsessive-Compulsive: Ps: Psychoticism; PI: Paranoid Ideation.

The numbers represent the standardized loadings beta estimates. Due to multicollinearity, the standardized loadings estimates can exceed the bounds of (-1,1) (Deegan Jr, 1978).

sTable 3: Factor loadings for a correlated-factors model of psychopathology with cognition

	Factor loadings			
	Ex	In	TD	IQ
VU	0	0	0	.850
VS	0	0	0	.831
MR	0	0	0	.797
Ca	0	0	0	.861
SA	.522	0	0	0
CD	.812	0	0	0
IS	.905	0	0	0
De	0	.977	0	0
An	0	.914	0	0
PA	0	.887	0	0
OC	0	0	.941	0
Ps	0	0	1.006	0
PI	0	0	.916	0

Note.

Abbreviations. Ex: Externalizing; In: Internalizing; TD: Thought Disorder; SA: Substance Abuse; CD: Conduct Disorder (i.e., Hostility); IS: Interpersonal Sensitivity; De: Depression; An: Anxiety; PA: Phobic Anxiety; OC: Obsessive-Compulsive: Ps: Psychoticism; PI: Paranoid Ideation; IQ: Intelligence Quotient; VU: Verbal Understanding; VS: Visual-Spatial problem-solving abilities; MR: Mathematical reasoning; Ca: Categorization abilities.

The numbers represent the standardized loadings beta estimates. Due to multicollinearity, the standardized loadings estimates can exceed the bounds of (-1,1) (Deegan Jr, 1978).

sTable 4: Factor loadings for a hierarchical model of psychopathology

Factor loadings

	Ex	In	TD	P
SA	.512	0	0	0
CD	.808	0	0	0
IS	.911	0	0	0
De	0	.978	0	0
An	0	.915	0	0
PA	0	.884	0	0
OC	0	0	.941	0
Ps	0	0	1.009	0
PI	0	0	.914	0
Ex	0	0	0	1.018
In	0	0	0	1.007
TD	0	0	0	1.023

Note.

Abbreviations. P: A general factor of psychopathology; Ex: Externalizing; In: Internalizing; TD: Thought Disorder; SA: Substance Abuse; CD: Conduct Disorder (i.e., Hostility); IS: Interpersonal Sensitivity; De: Depression; An: Anxiety; PA: Phobic Anxiety; OC: Obsessive-Compulsive: Ps: Psychoticism; PI: Paranoid Ideation.

The numbers represent the standardized loadings beta estimates. Due to multicollinearity, the standardized loadings estimates can exceed the bounds of (-1,1) (Deegan Jr, 1978).

sTable 5: Factor loadings for a hierarchical model of psychopathology with cognition

Factor loadings

	Ex	In	TD	IQ	P
VU	0	0	0	.849	0
VS	0	0	0	.831	0
MR	0	0	0	.800	0
Ca	0	0	0	.859	0
SA	.521	0	0	0	0
CD	.811	0	0	0	0
IS	.905	0	0	0	0
De	0	.977	0	0	0
An	0	.914	0	0	0
PA	0	.888	0	0	0
OC	0	0	.941	0	0
Ps	0	0	1.007	0	0
PI	0	0	.916	0	0
Ex	0	0	0	0	1.024
In	0	0	0	0	1.001
TD	0	0	0	0	1.027
IQ	0	0	0	0	-.253

Note.

Abbreviations. P: A general factor of psychopathology; Ex: Externalizing; In: Internalizing; TD: Thought Disorder; SA: Substance Abuse; CD: Conduct Disorder (i.e., Hostility); IS: Interpersonal Sensitivity; De: Depression; An: Anxiety; PA: Phobic Anxiety; OC: Obsessive-Compulsive: Ps: Psychoticism; PI: Paranoid Ideation; IQ: Intelligence Quotient; VU: Verbal Understanding; VS: Visual-Spatial problem-solving abilities; MR: Mathematical reasoning; Ca: Categorization abilities.

The numbers represent the standardized loadings beta estimates. Due to multicollinearity, the standardized loadings estimates can exceed the bounds of (-1,1) (Deegan Jr, 1978).

sTable 6: Factor loadings for a single factor model of psychopathology

Factor loadings

	P
SA	.520
CD	.822
IS	.928
De	.986
An	.921
PA	.888
OC	.953
Ps	1.023
PI	.926
P	0

Note.

Abbreviations. P: A general factor of psychopathology; Ex: Externalizing; In: Internalizing; TD: Thought Disorder; SA: Substance Abuse; CD: Conduct Disorder (i.e., Hostility); IS: Interpersonal Sensitivity; De: Depression; An: Anxiety; PA: Phobic Anxiety; OC: Obsessive-Compulsive: Ps: Psychoticism; PI: Paranoid Ideation.

The numbers represent the standardized loadings beta estimates. Due to multicollinearity, the standardized loadings estimates can exceed the bounds of (-1,1) (Deegan Jr, 1978).

sTable 7: Factor loadings for a single factor model of psychopathology with cognition

Factor loadings

	P
VU	-.237
VS	-.226
MR	-.241
Ca	-.236
SA	.530
CD	.829
IS	.925
De	.982
An	.917
PA	.891
OC	.954
Ps	1.021
PI	.928
P	0

Note.

Abbreviations. P: A general factor of psychopathology; Ex: Externalizing; In: Internalizing; TD: Thought Disorder; SA: Substance Abuse; CD: Conduct Disorder (i.e., Hostility); IS: Interpersonal Sensitivity; De: Depression; An: Anxiety; PA: Phobic Anxiety; OC: Obsessive-Compulsive: Ps: Psychoticism; PI: Paranoid Ideation; VU: Verbal Understanding; VS: Visual-Spatial problem-solving abilities; MR: Mathematical reasoning; Ca: Categorization abilities.

The numbers represent the standardized loadings beta estimates. Due to multicollinearity, the standardized loadings estimates can exceed the bounds of (-1,1) (Deegan Jr, 1978).

sTable 8: Fit indices for models of psychopathology with and without cognition: Sensitivity analysis of subpopulations

Model	RMSEA	TLI	CFI
**Top 20% of most symptomatic participants (N=245)**			
A correlated-factors model of psychopathology without cognition	0.080	0.888	0.895
A correlated-factors model of psychopathology with cognition	0.085	0.859	0.867
A hierarchical model of psychopathology without cognition	0.080	0.888	0.895
A hierarchical model of psychopathology with cognition	0.083	0.867	0.874
A single factor model of psychopathology without cognition	0.081	0.885	0.891
A single factor model of psychopathology with cognition	0.087	0.853	0.862
**Males (N=603)**			
A correlated-factors model of psychopathology without cognition	0.033	0.994	0.994
A correlated-factors model of psychopathology with cognition	0.042	0.989	0.989
A hierarchical model of psychopathology without cognition	0.033	0.993	0.994
A hierarchical model of psychopathology with cognition	-	-	-
A single factor model of psychopathology without cognition	0.033	0.994	0.994
A single factor model of psychopathology with cognition	-	-	-
**Females (N=586)**			
A correlated-factors model of psychopathology without cognition	0.036	0.992	0.992
A correlated-factors model of psychopathology with cognition	0.038	0.989	0.990
A hierarchical model of psychopathology without cognition	0.036	0.992	0.992
A hierarchical model of psychopathology with cognition	-	-	-
A single factor model of psychopathology without cognition	0.036	0.992	0.992
A single factor model of psychopathology with cognition	-	-	-
**Models based only on psychotic symptoms (Full sample; N=1189)**			
A correlated-factors model of psychopathology without cognition	0.020	0.997	0.998
A correlated-factors model of psychopathology with cognition	0.034	0.990	0.991
A hierarchical model of psychopathology without cognition	0.020	0.997	0.998
A hierarchical model of psychopathology with cognition	0.034	0.990	0.991
A single factor model of psychopathology without cognition	-	-	-
A single factor model of psychopathology with cognition	-	-	-

Note. Empty cells represent instances in which the model does not converge to a final solution. The goodness of fit was determined by the Tucker–Lewis Index (TLI) where values over 0.95 represent good fit, the Comparative Fit Index (CFI) where values over 0.95 represent good fit, and the Root-Mean-Square Error of Approximation (RMSEA), where values under 0.05 represent good fit.

References

Deegan Jr, J. (1978). On the occurrence of standardized regression coefficients greater than one. *Educational and Psychological Measurement, 38*(4), 873-888.
